# Malignant transformation of ovarian mature cystic teratoma with rupture, elevated serum CA199, CA12, CEA: A case report

**DOI:** 10.1097/MD.0000000000038793

**Published:** 2024-09-13

**Authors:** Liang Chen, Lijie Dong

**Affiliations:** a Department of Radiology, Binzhou Medical University Hospital, Binzhou, Shandong, P. R. China.

**Keywords:** imaging finding, malignant transformation, mature cystic teratoma, rupture, serum cancer antigen

## Abstract

**Rationale:**

Reports of mature cystic teratomas (MCTs) with associated complications and changes in serum cancer antigen levels are rare. Herein, we report a rare case of MCT with associated complications (rupture and malignant transformation), high levels of serum cancer antigens (CA19-9, CA12, and CEA), and surgical therapy.

**Patient concerns:**

An 81-year-old woman was referred to our emergency department because of diffuse abdominal pain and distension for 20 days.

**Diagnoses:**

Imaging findings, including transabdominal ultrasonography, computed tomography, and magnetic resonance imaging, revealed a complex solid cystic mass in the lower abdomen. Preoperative laboratory test results showed high levels of serum cancer antigens (CA19-9, CA12, and CEA) in MCT. Histopathological examination of the specimen revealed a MCT with rupture and malignant transformation.

**Interventions:**

The patient underwent a total abdominal hysterectomy, bilateral oophorectomy, and partial omentectomy. The patient did not undergo chemotherapy after surgery.

**Outcomes:**

The follow-up period was 12 months. The patient recovered well without focal local recurrence or distant metastasis after the surgery.

**Lessons:**

The study aims to report a new case of MCT with associated complications (rupture and malignant transformation) and changes in serum cancer antigen levels. Although this tumor presents as a complex solid cystic mass, detection of the intratumoral fat component is a key diagnostic imaging feature. A high level of serum cancer antigen may indicate the malignant transformation of MCT. In this case, surgery was an effective treatment for the MCT.

## 1. Introduction

Mature cystic teratomas (MCTs) are one of the most common ovarian tumors, originating from 3 germ layers (mesoderm, endoderm, and ectoderm).^[1]^ Although MCTs occur at any age (2–80 years with a mean age of 32 years), only 5% occur in postmenopausal women.^[2]^ Its clinical course is usually insidious, and its symptoms include abdominal pain, distention, and abdominal or pelvic mass or swelling, and it is often incidentally found on imaging.^[3]^ The main complication of MCT is cyst torsion (16%); other reported rare complications include malignant transformation (1–2%), infection (1%), and rupture (0.3–2%).^[4,5]^ Malignant changes of MCTs are rare, and the most common histological type is squamous cell carcinoma (SCC).^[5]^ Some tumor markers have been reported to be useful for MCT characterization and prediction of malignant transformation.^[6]^ Herein, we report a case of malignant transformation (SCC) of an ovarian MCT, chemical peritonitis caused by spontaneous rupture and changing preoperative serum cancer antigen levels (CA19-9, CA12, and CEA) in an 81-year-old woman. The objective of this report is to describe the imaging signs suggestive of MCT rupture and malignant transformation and discuss the clinical characteristics, imaging manifestations, preoperative serum cancer antigen levels, and treatment of this disease.

## 2. Case report

An 81-year-old woman (gravida 4, para 4) was admitted to the emergency department of Binzhou Medical Hospital on February 06, 2023, with diffuse abdominal pain and bloating for 20 days. In questioning the patient, we found that she experienced nausea, vomiting, and absent bowel sounds for 2 days, but she denied any traumatic history. The patient did not receive treatment before coming to the hospital.

Physical examination revealed that the patient’s vital signs were stable and temperature was normal. Abdominal examination revealed a distended abdomen with marked rebound tenderness in the lower abdomen.

Preoperative laboratory test results showed a white cell count of 17,100/mL, neutrophil 92.7%, C-reactive protein 63.40 mg/L, high CA199 levels (>1000 U/mL; normal range, <27 U/mL), high CA125 levels (224 U/mL; normal range, <35 U/mL), and high carcinoembryonic antigen (CEA) (73.4 U/mL; normal range, <3.4 U/mL).

**Figure 1.**
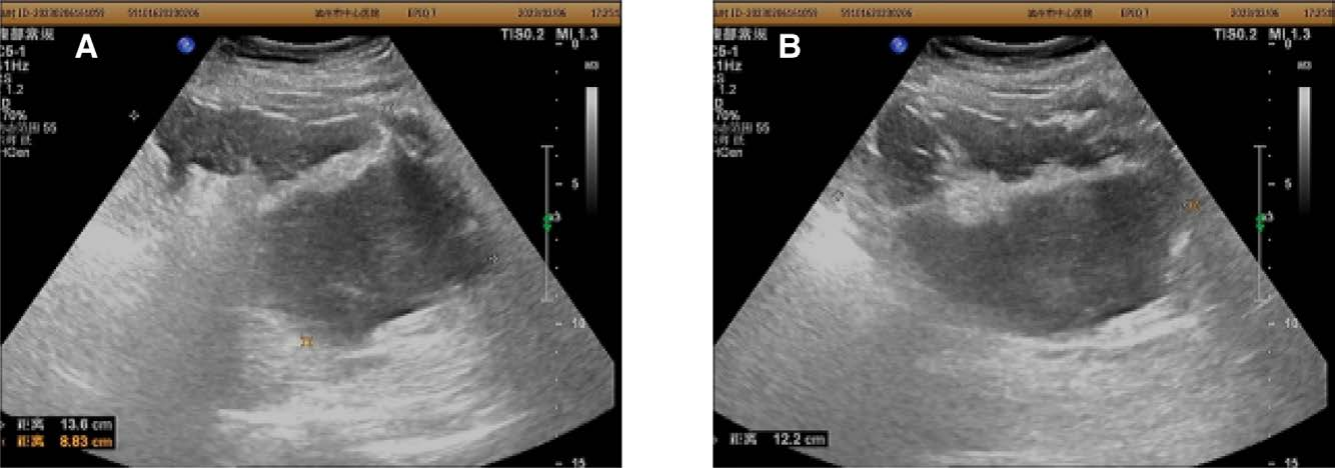
(A and B)Transabdominal ultrasonography showed a mixed echogenic masse measuring 12.2 × 13.6 × 8.3 cm in lower abdominal.

Transabdominal ultrasonography showed a mixed echogenic masse measuring 12.2 × 13.6 × 8.3 cm in the lower abdomen (Fig. 1).

**Figure 2.**
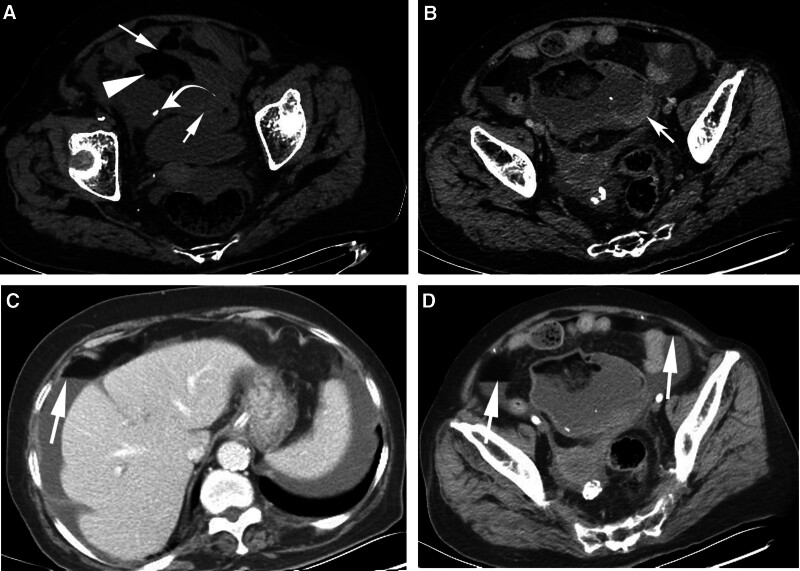
(A) CT scan showed predominantly cystic mass (arrowheads) in lower abdomen with disruptive tumor margin (thin arrows), irregular calcified entity (curved arrow), and fat contents (thick arrows). (B) Contrast-enhanced CT image shows enhanced solid components (thin arrow) of lesion. (C and D) Contrast-enhanced CT scan respectively shows ascites with scattered multiple fat-fluid levels (arrows; fat: <130 HU) in bilateral subphrenic regions and pelvic cavity.

Computed tomography (CT) demonstrated a huge heterogeneous mass measuring 11.7 × 9.3 cm in the pelvis, and presented with irregular solid components (Fig. 2A). Furthermore, fat and calcification were observed in the mass, and the tumor margins were disrupted (Fig. 2A). On contrast-enhanced CT, the solid components of the tumor were enhanced (Fig. 2B). The abdomen was filled with moderate amounts of ascites with thickened and multiple fat-fluid levels scattered in the abdominal cavity (Fig. 2C and D).

**Figure 3.**
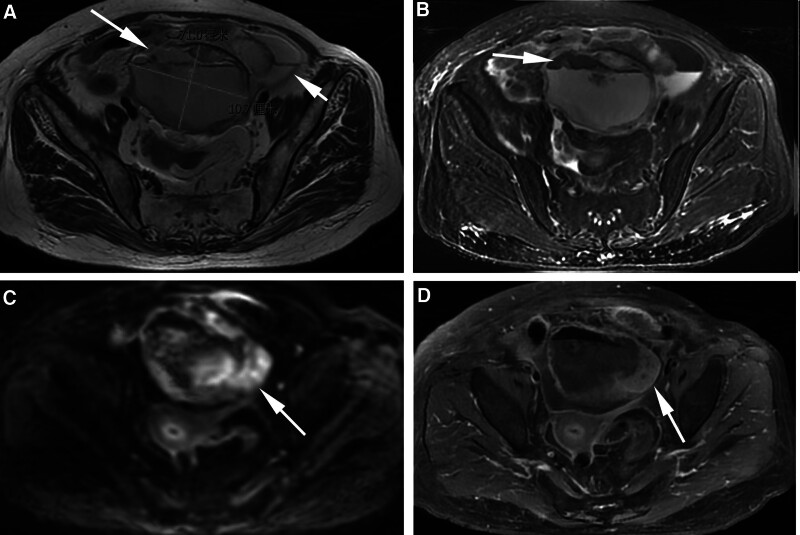
(A) Axial T2-weighted image shows cystic lesion (long arrow) with dimensions of 10.7 × 7.1 cm and ascites (short arrow). (B) Axial T2-weighted image with fat saturation shows low signal intensity of the supernatant fatty layer creating an interface with a high signal intensity aqueous layer (arrow). (C) Axial diffusion-weight (DWI) shows high signal intensity in a section of the tumor wall (arrow). (D) Axial T1-weighted image with fat suppression and contrast enhancement shows which the tumor wall was enhanced (arrow).

Magnetic resonance imaging (MRI) revealed a cystic mass measuring 10.7 × 7.1 cm. The tumor size was clearly reduced compared to that observed on the CT and ultrasonography images. Fat (Fig. 3A and B) and ascites (Fig. 3A) were observed. On diffusion weighted imaging, we observed a high signal intensity in a section of the tumor wall (Fig. 3C), which was enhanced (Fig. 3D). Considering the clinical symptoms, ultrasound, CT, and MRI findings, we suspected chemical peritonitis due to a ruptured MCT.

Operative findings revealed approximately 500 mL of ascites, including the adipose component. A mass of 11.5 cm in size was seen in the left ovary, which had already ruptured prior to abdominal surgery. Furthermore, the tumor strongly adhered to the greater omentum. The liver, spleen, bowel, stomach, uterus, and right ovary were normal. Intraoperative frozen sections showed well-differentiated neoplastic squamous cells with hyperchromatic and pleomorphic nuclei. The patient then underwent abdominal total hysterectomy, bilateral oophorectomy, and partial omentectomy.

**Figure 4.**
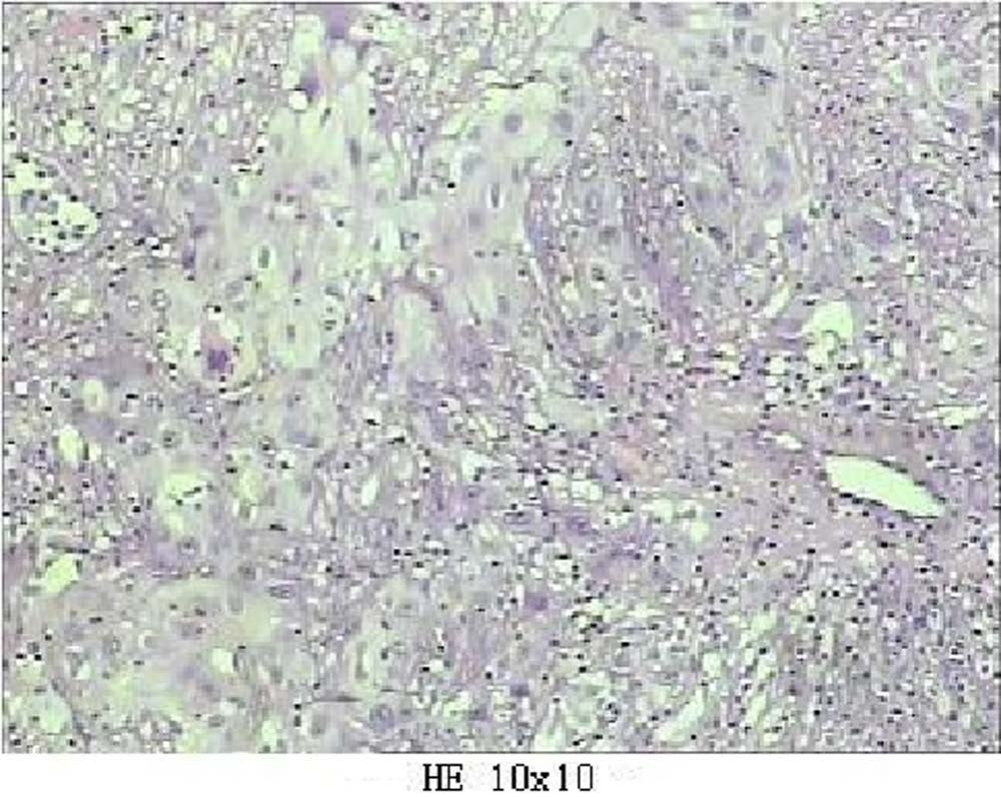
Squamous cell carcinoma arising in the mature cystic teratoma of the ovary (H&E, 10 × 10).

Histopathological examination of the specimen revealed a MCT with malignant transformation (Fig. 4). The solid components revealed malignant transformation of SCC. Malignant cells were not detected during the cytodiagnosis of ascites. The patient recovered uneventfully and was discharged on postoperative day 7. The follow-up period was 12 months. The patient was in good condition without any complications.

## 3. Discussion

MCT is a common disease among women, accounting for 20% of all ovarian neoplasm; however, MCT combined with multiple complications is rarely reported.^[7]^ The present case of MCT with associated multiple complications (rupture and malignant transformation).

Complications associated with MCTs include torsion (16%), malignant transformation (1–2%), rupture (1–2%), and infection (1%).^[4,5]^ In our case, the patient presented simultaneously with a common appearance and 2 rare complications (malignant transformation and rupture), making our patient unique from the previously presented case.

MCTs are defined as tumors composed of 3 germ cell lines (mesoderm, endoderm, and ectoderm), and the content of this cystic formation may include sebum, hair, skin, and occasionally teeth as well.^[1]^ Malignant transformation may occur in any of these 3 germ cell lines. More than 80% of secondary malignant transformation of ovarian teratoma are squamous cell carcinoma (SCC) emerging from the ectoderm, while the rest include a wide variety of malignancies such as adenocarcinoma, sarcoma, carcinoid, and melanoma.^[7,8]^ Useful clinical factors to raise suspicions for malignant transformation include concentration level of tumor markers, patient age, and the maximum diameter of the tumor.^[8]^

Malignant transformation of mature ovarian teratomas usually occurs in postmenopausal women, that is, those in their fifth or sixth decade of life.^[2]^ In contrast, benign cystic teratomas usually occur in women of reproductive age. One study comparing benign and SCC MCTs reported an average of 32.7 years old and 50.8 years, respectively.^[9]^ Our patient, at 81 years of age, supports the notion that SCC arising in MCTs occurs more commonly in older patients.

One report suggested that tumor size is the most important factor for the differential diagnosis between benign and malignant MCTs.^[8,10]^ According to some studies, tumors larger than 9.9 cm are associated with an increased risk of MCT,^[8]^ and tumors larger than 15 cm are associated with more aggressive behavior.^[11]^ In our study, although the tumor was ruptured, its size (maximum diameter 13.6 cm) was greater than the cutoff.

Soft tissue formation in an MCT, namely, the Rokitansky nodule or dermoid plug, is commonly observed, but benign teratomas never show growth of wall nodules into the cavity.^[12]^ Malignant transformation normally originates from the Rokitansky nodule or dermoid plugs. Therefore, when we observe the growth of large irregularly marginated soft tissue lesions at the wall of or within the tumor on CT or MR, we should raise suspicion about the malignancy of the tumor, and the appropriate sections must be selected and analyzed during histopathological investigation.^[1,12]^ In our patient, we found solid components of the cystic wall on enhanced MRI and CT, and high signal intensity in the solid components of the tumor wall on DW-MRI.

Several studies have investigated the diagnostic value of serum tumor markers in cases of malignant transformation of MCT. One report suggested that increased serum CA199 levels are a risk factor for malignant transformation of MCT.^[6]^ Kim et al revealed that elevated preoperative serum CA125 may have prognostic value in patients with malignant transformation of MCT.^[13]^ One study concluded that high level of CEA was useful marker for the diagnosis of MTC.^[14]^

Spontaneous rupture of teratomas can occur, but are rare, due to thick cystic walls, with the possibility of occurrence ranging between 0.3% and 0.7%.^[15]^ The exact causes of rupture are mostly unknown, but hypothetical causes of tumor rupture include torsion with tumor infarction, direct trauma, prolonged pressure from the fetus during pregnancy, delivery, infection of dermoid contents, malignant change, increased internal pressure due to rapid growth of the cyst, and tumor size.^[16]^ In our case, a disruptive tumor margin was observed on MRI. To the best of our knowledge, there is tumor rupture due to a malignant-transformed ovarian cystic teratoma. MCT may rupture into the peritoneal cavity, and leakage or spillage of MCT contents can cause acute or chronic chemical peritonitis, which depends on the leakage time and is an aseptic inflammatory peritoneal reaction.^[13]^ Chemical peritonitis can result in pelvic adhesive disease, bowel obstruction, abdominal wall abscesses, enterocutaneous fistula formation, and other complications.^[17]^ The fat-fluid level seen on CT or MRI suggest rupture of MCT,^[1,17]^ as seen in our patient. The clinical presentation might have been insignificant in the early period; however, the patient complained of progressive abdominal pain, abdominal distention, fever, and gastrointestinal disturbances such as anorexia, nausea, vomiting, and diarrhea.^[8]^ When an MCT ruptures spontaneously, an emergency operation is usually performed and peritonitis is alleviated by irrigating the abdominal cavity. The number of patients with malignant transformation of MCTs is small and there are insufficient data for clear guidance on optimal management strategies. Total abdominal hysterectomy, bilateral salpingo-oophorectomy, and omentectomy are generally accepted treatment approaches for SCC patients.^[8]^ Thorough saline wash has been suggested for preoperative or perioperative ruptures to reduce complications, such as chemical peritonitis, intra-abdominal adhesions, and masses.^[2]^ Unlike most ruptures due to laparoscopic operations, our patient’s rupture occurred preoperatively, as demonstrated by CT and MRI, and was subsequently confirmed by pathologic evaluation.

## 4. Conclusion

Malignant transformation and rupture of MCTs are very rare complications. High levels of serum cancer antigens (CA199, CA125, and CEA) may indicate the malignant transformation of MCT. Familiarization with the presentation, imaging, and microscopic features of MCT can improve the timeliness and accuracy of diagnosis, disease management, and outcomes in patients with ovarian MCT.

## Author contributions

**Writing – original draft:** Liang Chen.

**Writing – review & editing:** Lijie Dong.
